# Polydatin Attenuates Sepsis-Induced Lung Injury by Inhibiting Neutrophil Extracellular Traps Formation via Nrf2/HO-1 Pathway

**DOI:** 10.3390/biomedicines14040827

**Published:** 2026-04-05

**Authors:** Hongkai Dai, Cheng Li, Bin Yang, Qianrui Huang, Xiao Ran, Yingfang Zheng, Yongsheng Li

**Affiliations:** 1 Department of Emergency Medicine, Tongji Hospital, Tongji Medical College, Huazhong University of Science and Technology, Wuhan 430000, China; 2 Department of Intensive Care Medicine, Tongji Hospital, Tongji Medical College, Huazhong University of Science and Technology, 1095 Jiefang Road, Wuhan 430000, China

**Keywords:** polydatin, sepsis, lung injury, neutrophil extracellular traps, Nrf2/HO-1

## Abstract

**Background:** Sepsis-induced acute lung injury (ALI) is a life-threatening condition with limited therapeutic options. Neutrophil extracellular traps (NETs) contribute to its pathogenesis. This study investigated whether polydatin (PD) protects against septic ALI by inhibiting NETs via the Nrf2/HO-1 pathway. **Methods:** A cecal ligation and puncture (CLP)-induced septic ALI mouse model and an LPS-stimulated neutrophil model were established. Lung injury was assessed by histology, lung wet/dry ratio, BALF protein, and inflammatory cytokines. Endothelial junction proteins and NETs markers were examined by Western blot, immunofluorescence, and SYTOX Green staining. Nrf2/HO-1 pathway activation and ML385 inhibitor experiments were performed for mechanistic validation. **Results:** PD dose-dependently attenuated lung injury, preserved endothelial junction proteins (ZO-1, VE–cadherin, occludin), and suppressed NETs formation in vivo. In vitro, PD activated Nrf2/HO-1, promoted Nrf2 nuclear translocation, reduced ROS, and inhibited LPS-induced NETs. These effects were abrogated by ML385, confirming pathway specificity. **Conclusions:** PD mitigates septic ALI by inhibiting NETs formation. In vitro mechanistic studies further suggest that this effect is mediated through activation of the Nrf2/HO-1 antioxidant pathway, positioning PD as a potential therapeutic candidate for sepsis-induced ALI.

## 1. Introduction

Sepsis-induced acute lung injury (ALI) is one of the most common complications of sepsis, characterized by high morbidity and mortality rates, and has become a major global health burden [1,2]. The pathogenesis of sepsis-induced ALI involves complex mechanisms, including excessive inflammatory response, oxidative stress, endothelial barrier disruption, and neutrophil-mediated tissue damage [3,4]. Despite decades of research, effective pharmacological interventions for sepsis-induced ALI remain limited, and current treatment strategies primarily rely on supportive care [5,6]. Therefore, effective pharmacological agents for sepsis-induced ALI are urgently needed.

Neutrophils play a critical role in the pathogenesis of septic ALI [7,8]. Upon activation, neutrophils release neutrophil extracellular traps (NETs), which are web-like structures composed of decondensed chromatin coated with granular proteins, such as citrullinated histone H3 (CitH3) and myeloperoxidase (MPO) [9,10]. Although NETs are essential for trapping and killing pathogens, excessive NET formation has been implicated in tissue damage, endothelial barrier disruption, and inflammatory amplification during sepsis [7]. Accumulating evidence has demonstrated that NETs contribute to septic ALI by directly injuring pulmonary endothelial cells and promoting vascular permeability [9,11]. The formation of NETs is critically dependent on the production of reactive oxygen species (ROS), and studies have shown that ROS scavengers can significantly prevent NETs release [12,13]. Therefore, targeting NET formation represents a promising therapeutic strategy for septic ALI. The nuclear factor erythroid 2-related factor 2 (Nrf2) is a master transcription factor that regulates antioxidant responses by promoting the expression of heme oxygenase-1 (HO-1) and other cytoprotective enzymes [14,15]. By reducing cellular ROS levels, the Nrf2/HO-1 pathway has been shown to protect against various lung injuries [16,17]. Importantly, activation of the Nrf2/HO-1 pathway has been reported to inhibit NETosis in several disease models by reducing intracellular ROS levels [18]. These findings suggest that enhancing Nrf2/HO-1-mediated antioxidant capacity may suppress excessive NETs formation and alleviate septic ALI.

Polydatin (PD) is a natural stilbenoid compound derived from the traditional Chinese herb *Polygonum cuspidatum*. This compound has been reported to possess diverse pharmacological properties, including anti-inflammatory, antioxidant, and anti-apoptotic effects [19,20,21]. Studies have shown that PD attenuates LPS-induced acute lung injury by inhibiting inflammatory responses and oxidative stress [22,23]. Moreover, PD has been demonstrated to activate the Nrf2/HO-1 pathway in various disease models, thereby exerting cytoprotective effects [24,25,26]. Given that *Polygonum cuspidatum* has been traditionally used for its detoxifying and anti-inflammatory properties, we wondered whether PD could inhibit NET formation during sepsis. A recent study showed that PD alleviated severe traumatic brain injury-induced acute lung injury by inhibiting S100B-mediated NET formation [27]. Additionally, PD has been reported to suppress NET release in lupus-prone mouse models and rheumatoid arthritis by reducing ROS production [28,29]. However, the effect of PD on sepsis-induced NET formation has not been reported. More importantly, while PD is known to activate the Nrf2/HO-1 pathway, whether this antioxidant mechanism is directly responsible for its inhibition of NETosis in any disease model remains unexplored. Therefore, this study aimed to investigate not only if PD attenuates sepsis-induced ALI by inhibiting NET formation, but critically, to test the causal hypothesis that this effect is mediated through activation of the Nrf2/HO-1 antioxidant pathway.

## 2. Materials and Methods

### 2.1. Reagents

The antibodies used in the study are shown in Table 1. The reagents used in this study are shown in Table 2.

### 2.2. Animals and Treatment

**Figure 1:**
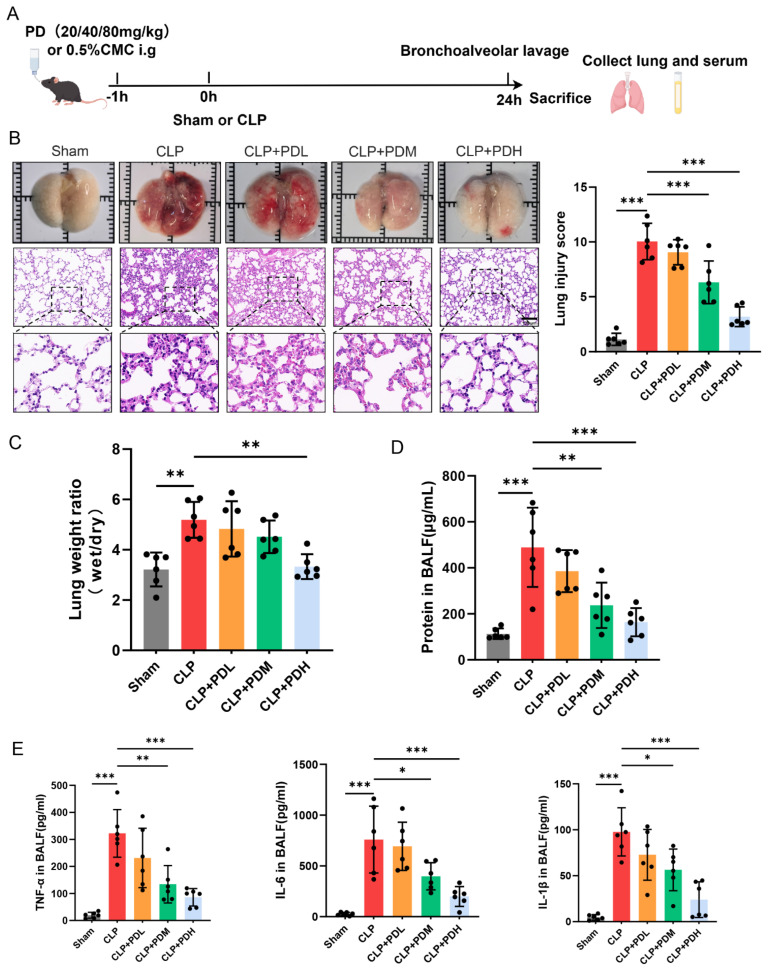
PD attenuates sepsis-induced lung injury by reducing pulmonary inflammation and permeability in mice. (**A**) Animal experimental protocol. (**B**) Representative photographs of gross lung morphology, H&E staining, and lung injury scores (*n* = 6), scale bar = 100 µm. (**C**) Wet-to-dry weight ratio in lung tissue (*n* = 6). (**D**) Total protein concentration in BALF (*n* = 6). (**E**) Levels of inflammatory cytokines TNF-α, IL-6, and IL-1β in BALF, measured by ELISA (*n* = 6). * *p* < 0.05, ** *p* < 0.01, and *** *p* < 0.001.

Wild-type C57BL/6J mice (8-week-old males, weighing 23–25 g) were purchased from Beijing Vital River Laboratory Animal Technology Co., Ltd. (Beijing, China). All animals were housed under specific pathogen-free (SPF) conditions at 20 ± 2 °C and 60 ± 5% humidity, with a 12 h light/dark cycle, and with free access to food and water. After one week of adaptation, the mice were randomly divided into 5 groups: Sham group (*n* = 6), CLP group (*n* = 12), CLP + PDL (20 mg/kg) group (*n* = 12), CLP + PDM (40 mg/kg) group (*n* = 12) and CLP + PDH (80 mg/kg) group (*n* = 12). The doses of polydatin were selected based on previously published studies demonstrating its protective effects and safety profile in murine models of acute lung injury and inflammation [22]. Polydatin was administered via oral gavage 1 h prior to CLP surgery. The sham and CLP groups received an equivalent volume of vehicle (0.5% carboxymethyl cellulose, CMC). At 24 h after the operation, the mice in each group were anesthetized and sacrificed. The experimental scheme is shown in Figure 1A. All experimental procedures were reviewed and approved by the Animal Ethics Committee of Tongji Hospital, Tongji Medical College, Huazhong University of Science and Technology (Approval No. TJH-202410004).

Sepsis was triggered using a method involving cecal ligation and puncture (CLP), as outlined in previous research [30]. In summary, the mice were placed under anesthesia with isoflurane, and a midline laparotomy was conducted. The distal third of the cecum was ligated and then punctured once with a 21-gauge needle. To confirm the puncture site was open, a small amount of fecal material was expelled, after which the cecum was repositioned in the abdominal cavity, and the incision was carefully closed in layers. To support recovery, all mice received 1 mL of warm saline subcutaneously for fluid resuscitation and meloxicam (5 mg/kg) for pain relief post-surgery. The mice that underwent sham surgery experienced the same surgical steps, but without any ligation or puncture.

#### Murine Sepsis Score

The Murine Sepsis Score (MSS) was used for longitudinal assessment of sepsis severity. This scoring system aggregates seven clinical parameters: appearance, level of consciousness, activity, response to stimulus, eye appearance, respiratory rate, and respiratory quality. Each parameter is graded on a scale of 0 to 4, where 0 represents normal, 1 represents mild impairment, 2 represents moderate impairment, 3 represents severe impairment, and 4 represents moribund or dead. The scores for all seven parameters are summed to generate a total MSS, with a maximum score of 28. Higher scores indicate more severe sepsis [31].

### 2.3. Lung Wet-to-Dry Weight Ratio

To assess the degree of pulmonary edema, the wet-to-dry weight ratio (W/D) of lung tissue was measured. The lung tissue was removed, surface blood was wiped off, and the wet weight was immediately recorded. The tissue was then placed in a 60 °C oven for 72 h until a constant dry weight was achieved, and the dry weight value was recorded. The wet-to-dry weight ratio was calculated by dividing the wet weight by the dry weight.

### 2.4. BALF Collection and Total Protein Assay

Bronchoalveolar lavage fluid (BALF) was collected from the mice 24 h post-surgery. After tracheal intubation, lungs were lavaged three times with 1 mL of ice-cold PBS containing protease inhibitors. The lavage fluid was centrifuged (SciLogex, Rocky Hill, CT, USA) at 500 *g* for 10 min at 4 °C. The supernatant was collected, and total protein concentration was measured using a BCA protein assay kit following the manufacturer’s instructions. Absorbance was read at 562 nm, and protein concentrations were calculated against a bovine serum albumin (BSA) standard curve.

### 2.5. Enzyme-Linked Immunosorbent Assay

Concentrations of the pro-inflammatory cytokines IL-6, TNF-α, and IL-1β in BALF were quantified using commercial mouse-specific ELISA kits (MultiSciences (Lianke) Biotech, Hangzhou, China), following the manufacturer’s protocols. Standard curves were used to determine cytokine levels.

### 2.6. Isolation and Stimulation of Mouse Bone Marrow Neutrophils

Neutrophils were purified from mouse bone marrow using Percoll density gradient centrifugation (SciLogex, Rocky Hill, CT, USA) [31]. In brief, cells flushed from femurs and tibias were layered onto a discontinuous Percoll gradient (65%/80%). After centrifugation at 1000 *g* for 30 min at room temperature, the neutrophil fraction was recovered from the 65%/80% interface. Isolated cells were rested in culture medium for 1 h prior to stimulation with LPS (10 μg/mL) or the vehicle for 4 h, then harvested for downstream assays.

### 2.7. Cell Culture and Treatment

Human umbilical vein endothelial cells (HUVECs) were obtained from Procell Co., Ltd. (Wuhan, China) and cultured in endothelial cell medium (ECM) supplemented with 10% fetal bovine serum (FBS), 1% endothelial cell growth supplement, and 1% penicillin/streptomycin at 37 °C in a humidified cell culture incubator with 5% CO_2_. Bone marrow-derived neutrophils were freshly isolated from the mice as described above and cultured in RPMI 1640 medium (Gibco, Waltham, MA, USA) supplemented with 10% FBS and 1% penicillin/streptomycin.

For in vitro experiments, cells were treated with LPS in the presence or absence of PD (10 μM) for 4 h. In inhibitor experiments, cells were pre-incubated with ML385 (10 μM) for 1 h prior to LPS and PD stimulation.

### 2.8. Cell Viability Assay

According to the manufacturer’s protocol, the Cell Counting Kit-8 assay (Solarbio, Beijing, China) was used to test the effects of PD, ML385, and LPS + PD on cell viability.

### 2.9. Transwell Co-Culture Assay

Neutrophils and HUVECs were used in a transwell co-culture system to assess neutrophil-mediated endothelial injury. Briefly, HUVECs were seeded into the lower chamber of 24-well transwell plates and cultured to confluence. Neutrophils were then seeded into the upper chamber at a density of 5 × 10^5^ cells per well. The cells were co-cultured for 4 h in the presence or absence of LPS and/or PD. After incubation, HUVECs from the lower chamber were collected for Western blot or fixed for immunofluorescence staining.

### 2.10. Reactive Oxygen Species (ROS) Detection

Intracellular ROS levels were measured using the fluorescent probe 2′,7′-dichlorodihydrofluorescein diacetate (DCFH-DA). Briefly, neutrophils were seeded in 6-well plates and treated as indicated. After stimulation, the cells were incubated with 10 μM DCFH-DA in serum-free medium at 37 °C for 30 min in the dark. The cells were then washed three times with PBS to remove excess probe. Fluorescence images were captured using a fluorescence microscope, and mean fluorescence intensity was quantified using ImageJ software (v1.54, National Institutes of Health, Bethesda, MD, USA).

### 2.11. H&E Staining

Lung tissues were fixed in 4% paraformaldehyde, embedded in paraffin, and cut into 5 μm sections. The sections were stained with hematoxylin and eosin (H&E), following standard protocols. Histopathological changes were examined under a light microscope by two independent pathologists blinded to the experimental groups. For each mouse, five randomly selected fields from three non-consecutive lung sections were examined at 200× magnification. Lung injury was scored based on the severity of four parameters: alveolar congestion, hemorrhage, leukocyte infiltration, and alveolar wall thickening. Each parameter was graded on a scale of 0 to 4, where 0 = no injury, 1 = mild (<25% involvement), 2 = moderate (25–50% involvement), 3 = severe (50–75% involvement), and 4 = very severe (>75% involvement). The scores for all four parameters were summed to generate a total lung injury score for each field, with a maximum score of 16. The average total score from all fields was calculated for each animal and used for statistical analysis, as previously described [32].

### 2.12. Immunohistochemistry

Lung tissue sections embedded in paraffin were deparaffinized and rehydrated. Heat-induced antigen retrieval was performed in citrate buffer (pH 6.0). To block endogenous peroxidase, the sections were treated with 3% H_2_O_2_ in methanol for 10 min at room temperature. Following overnight incubation at 4 °C with anti-Ly6G primary antibody, the sections were incubated with HRP-conjugated secondary antibody for 1 h at room temperature. Immunostaining was developed with DAB substrate, and the sections were counterstained with hematoxylin. Images were acquired using a light microscope and analyzed with ImageJ software.

### 2.13. Immunofluorescence Staining

Lung tissue sections and cells on coverslips were fixed in 4% paraformaldehyde for 15 min, permeabilized with 0.1% Triton X-100 (Solarbio, Beijing, China) for 15 min, and blocked with 5% BSA for 1 h. The samples were incubated overnight at 4 °C with primary antibodies, followed by fluorescence-labeled secondary antibodies for 1 h at room temperature in the dark. Nuclei were counterstained with DAPI for 5 min, and coverslips were mounted with antifade medium. Images were captured using a fluorescence microscope and analyzed with ImageJ software.

### 2.14. Western Blot

Protein samples were separated by SDS-PAGE and transferred onto 0.22 μm nitrocellulose membranes. The membranes were blocked with 5% non-fat milk for 1 h at room temperature and incubated overnight at 4 °C with primary antibodies. After washing, the membranes were incubated with appropriate HRP-conjugated secondary antibodies for 1 h at room temperature. Protein bands were visualized using a GeneGnome XRQ Chemiluminescence Imaging System (Synoptics Group, Frederick, MD, USA). Band intensities were quantified using ImageJ software and normalized to β-actin.

### 2.15. Statistical Analysis

Statistical analyses were performed using GraphPad Prism 9.0 (GraphPad Software, San Diego, CA, USA). Data are presented as the mean ± SD. Normality was assessed by the Shapiro–Wilk test. For multiple group comparisons, one-way ANOVA with Tukey’s post hoc test was used when assumptions of normality and homogeneity of variance were met. For comparisons against a single control group, one-way ANOVA with Dunnett’s post hoc test was applied. For non-normally distributed data, the Kruskal–Wallis test, followed by Dunn’s post hoc test, was used. Survival data were analyzed by the Kaplan–Meier method with the log-rank test. All experiments were independently repeated at least three times. Statistical significance was set at *p* < 0.05.

## 3. Results

### 3.1. Subsection

#### 3.1.1. PD Attenuates Sepsis-Induced Lung Injury by Reducing Pulmonary Inflammation and Permeability in Mice

To evaluate the protective effect of PD on sepsis-induced ALI, we established a CLP mouse model. Survival analysis revealed that the 24 h survival rate in the CLP group was 58.3%. PD treatment improved survival in a dose-dependent manner: the low dose did not improve survival, the medium dose increased survival to 66.7%, and the high dose significantly improved survival to 83.3%. Kaplan–Meier analysis with the log-rank test confirmed that the high-dose PD group had significantly higher survival compared to the CLP group (Figure S1A). Clinical severity was assessed using the MSS. The CLP mice exhibited significantly elevated MSS compared to the Sham mice, indicating severe sepsis. PD treatment dose-dependently reduced MSS, with the high-dose group showing the most pronounced improvement (Figure S1B). Compared with the Sham group, the CLP challenge resulted in severe lung damage, characterized by hemorrhage and edema. Histological examination revealed that CLP induced marked pulmonary pathological changes, including alveolar wall thickening, inflammatory cell infiltration, and interstitial edema, resulting in a significantly elevated lung injury score. Administration of PD attenuated these histopathological alterations in a dose-dependent manner. Specifically, treatment with medium or high doses of PD significantly reduced the lung injury score compared to the CLP group (Figure 1B). Consistently, PD administration reduced the lung W/D ratio (Figure 1C) and the total protein concentration in BALF (Figure 1D), indicating significant attenuation of pulmonary edema and alveolar–capillary barrier permeability. Furthermore, the CLP challenge markedly elevated the levels of pro-inflammatory cytokines, including TNF-α, IL-6, and IL-1β, in the BALF. The medium and high doses of PD significantly reduced the levels of cytokines compared to the CLP group (Figure 1E). These results demonstrate that PD mitigates sepsis-induced ALI by reducing pulmonary inflammation and vascular permeability, with medium and high doses exhibiting more pronounced protective effects.

#### 3.1.2. PD Attenuates Sepsis-Induced Lung Endothelial Barrier Disruption in Mice

**Figure 2:**
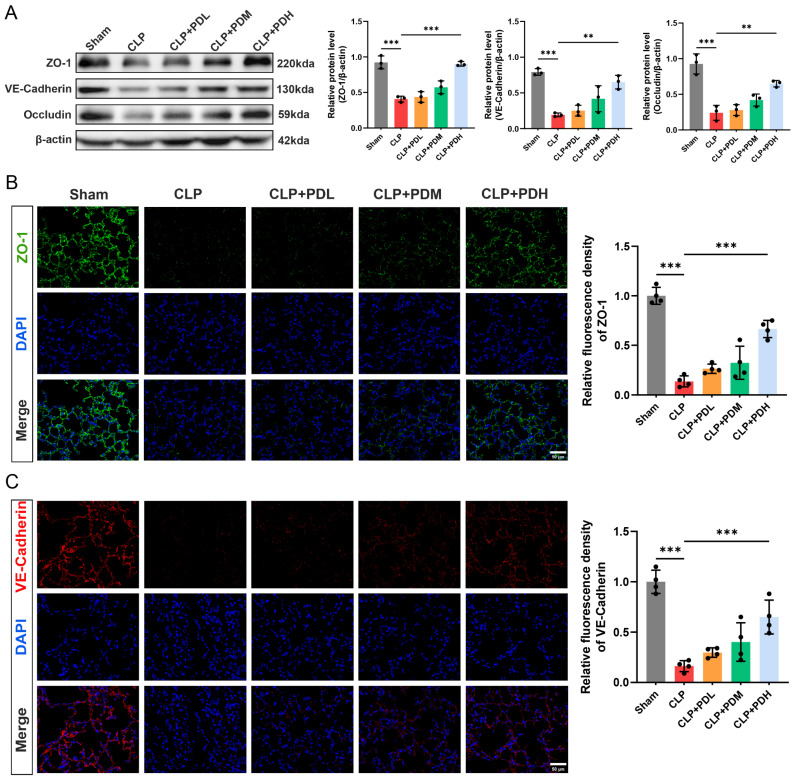
PD attenuates sepsis-induced lung endothelial barrier disruption in mice. (**A**) Representative Western blots and quantitative analysis of ZO-1, VE–cadherin, and occludin protein levels in lung tissues (*n* = 3). (**B**) Representative immunofluorescence staining of ZO-1 (green) in lung tissue sections. Nuclei were counterstained with DAPI (blue), and quantitative analysis of ZO-1 fluorescence intensity (*n* = 4) was performed, scale bar = 50 μm. (**C**) Representative immunofluorescence staining of VE–cadherin (red) in lung tissue sections. Nuclei were counterstained with DAPI (blue), and quantitative analysis of VE–cadherin fluorescence intensity (*n* = 4) was performed, scale bar = 50 μm. ** *p* < 0.01, and *** *p* < 0.001.

The above study has demonstrated that PD exhibits significant anti-inflammatory effects and effectively alleviates sepsis-induced lung injury. To investigate whether PD protects against lung endothelial barrier disruption, we examined the expression of tight junction proteins and the adherens junction proteins in lung tissues. Western blot analysis revealed that the CLP challenge markedly downregulated the protein levels of ZO-1, VE–cadherin, and occludin compared with the Sham group, and administration of high doses of PD significantly upregulated the levels of all three proteins compared to the CLP group (Figure 2A). To further visualize the distribution and expression of tight junction and adherens junction proteins, immunofluorescence staining was performed. ZO-1 immunostaining was predominantly localized at the cell borders of alveolar epithelial and endothelial cells in the Sham group, displaying a continuous and intact pattern. In contrast, the CLP challenge disrupted this integrity, resulting in fragmented and diminished ZO-1 fluorescence intensity. Quantitative analysis revealed that only the high dose of PD significantly restored ZO-1 fluorescence intensity compared to the CLP group, whereas the medium dose showed an increasing trend without statistical significance (Figure 2B). Similarly, VE–cadherin immunofluorescence staining exhibited a continuous linear pattern along the vascular endothelium in the Sham group, which was markedly disrupted in the CLP group, as evidenced by reduced and discontinuous staining (Figure 2C). Treatment with high doses of PD significantly preserved VE–cadherin fluorescence intensity compared to the CLP group (Figure 2C). These results demonstrate that PD protects against sepsis-induced lung endothelial barrier disruption by upregulating tight junction and adherens junction proteins, with high doses exhibiting more pronounced protective effects.

#### 3.1.3. PD Inhibited Neutrophil Infiltration and NET Formation in the Lungs of CLP Mice

**Figure 3:**
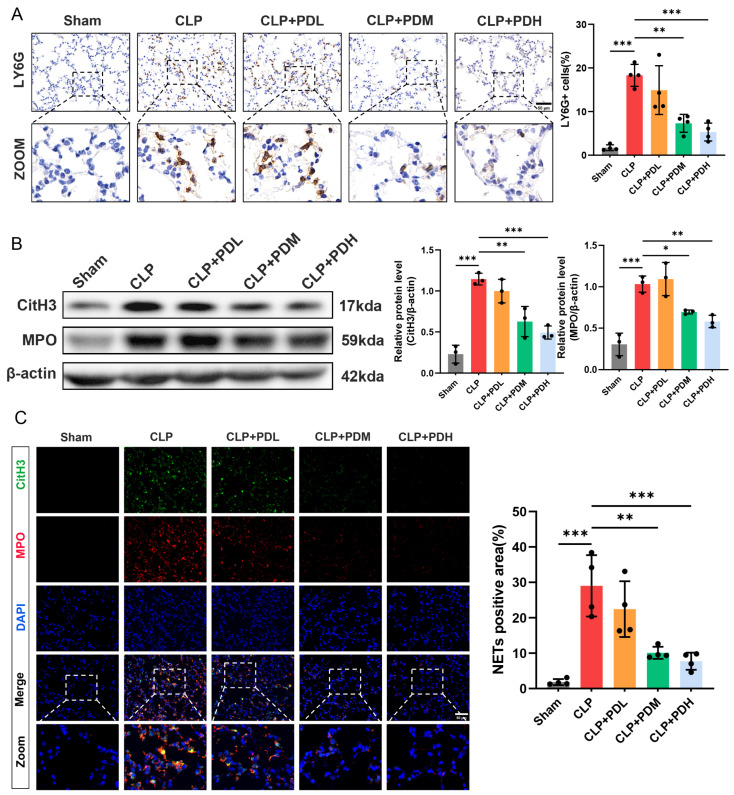
PD inhibited neutrophil infiltration and NET formation in the lungs of the CLP mice. (**A**) Representative immunohistochemical staining of Ly6G in lung tissue sections and quantitative analysis of Ly6G-positive cells (*n* = 4), scale bar = 50 μm. (**B**) Representative Western blots and quantitative analysis of CitH3 and MPO protein levels in lung tissues (*n* = 3). (**C**) Representative immunofluorescence co-staining of CitH3 (green) and MPO (red) in lung tissue sections. The nuclei were counterstained with DAPI (blue), and quantitative analysis of CitH3 and MPO double-positive areas (*n* = 4) was performed, scale bar = 50 μm. * *p* < 0.05, ** *p* < 0.01, and *** *p* < 0.001.

Neutrophil infiltration and NET formation are key contributors to sepsis-induced lung injury. To determine whether the protective effect of PD involves the regulation of these processes, we examined the expression of the neutrophil marker Ly6G and the NET-associated proteins CitH3 and MPO in lung tissues. Immunohistochemical staining revealed that Ly6G-positive neutrophils were rarely detected in the lungs of the Sham-operated mice. The CLP challenge markedly increased the infiltration of Ly6G-positive neutrophils into lung tissues. PD treatment dose-dependently reduced neutrophil infiltration, with medium and high doses significantly decreasing the number of Ly6G-positive cells compared to the CLP group (Figure 3A). Western blot analysis demonstrated that the CLP challenge significantly upregulated the protein levels of CitH3 and MPO compared with the Sham group. Consistent with the neutrophil infiltration results, administration of medium or high doses of PD significantly downregulated CitH3 and MPO expression compared to the CLP group (Figure 3B). To further visualize NET formation, immunofluorescence co-staining with CitH3 and MPO was performed on lung tissue sections. The CLP challenge induced abundant CitH3 and MPO co-localization, indicating the presence of NETs, which were rarely observed in the Sham group. Quantitative analysis of CitH3 and MPO double-positive areas revealed that medium and high doses of PD significantly reduced NET formation compared to the CLP group (Figure 3C). These results demonstrate that PD attenuates sepsis-induced lung injury by inhibiting neutrophil infiltration and NET formation, with medium and high doses exerting significant protective effects.

#### 3.1.4. PD Inhibits LPS-Induced NET Formation in Mouse Bone Marrow-Derived Neutrophils

**Figure 4:**
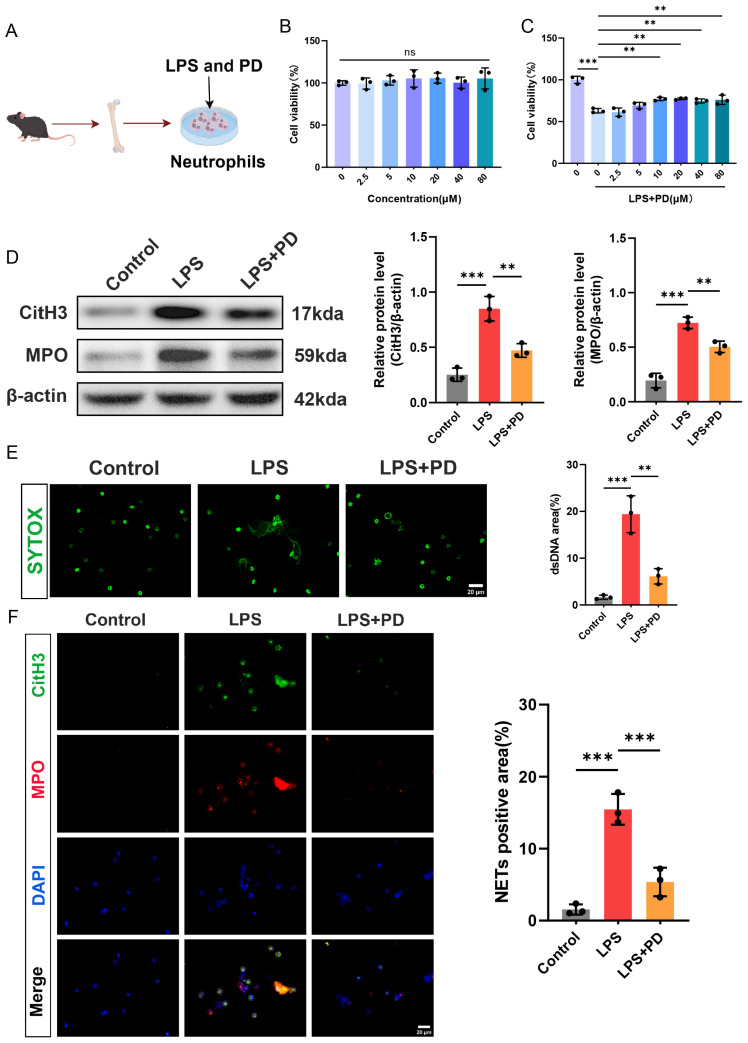
PD directly inhibits LPS-induced NET formation in mouse bone marrow-derived neutrophils. (**A**) The in vitro experimental protocol for this part. (**B**) The neutrophils were treated with increasing concentrations of PD (0–80 μM) for 4 h, and cell viability was measured by CCK-8 assay (*n* = 3). (**C**) The neutrophils were stimulated with LPS (10 μg/mL) in the presence or absence of increasing concentrations of PD (0–80 μM) for 4 h. Cell viability was measured by CCK-8 assay (*n* = 3). (**D**) Representative Western blots and quantitative analysis of CitH3 and MPO protein levels in neutrophils (*n* = 3). (**E**) Representative fluorescence images and quantitative analysis of SYTOX Green fluorescence intensity (*n* = 3), Scale bar = 20 µm. (**F**) Representative immunofluorescence co-staining of CitH3 (green) and MPO (red) in neutrophils. The nuclei were counterstained with DAPI (blue), and quantitative analysis of CitH3 and MPO double-positive areas (*n* = 3) was performed, scale bar = 20 μm. ** *p* < 0.01, and *** *p* < 0.001. ns = not statistically significant.

The animal experiments demonstrated the protective effect of PD on pulmonary endothelial injury and its inhibitory effect on the formation of NETs in lung tissue. To investigate whether PD directly suppresses NET formation in vitro, we isolated bone marrow-derived neutrophils from mice and stimulated them with LPS to induce NETosis (Figure 4A). First, to assess the cytotoxicity of PD on neutrophils and determine the optimal concentration for subsequent experiments, neutrophils were treated with increasing concentrations of PD (0, 2.5, 5, 10, 20, 40, and 80 μM) for 4 h, and cell viability was measured by CCK-8 assay. PD treatment alone at concentrations ranging from 2.5 to 80 μM did not affect neutrophil viability compared to the untreated control, indicating that PD is not toxic to neutrophils within this concentration range (Figure 4B). Next, neutrophils were stimulated with LPS (10 μg/mL) in the presence or absence of varying concentrations of PD (0, 2.5, 5, 10, 20, 40, and 80 μM) for 4 h. The CCK-8 assay revealed that LPS stimulation significantly reduced neutrophil viability to approximately 60% of the control level. PD treatment dose-dependently rescued LPS-induced cell death. Notably, 10 μM of PD significantly increased cell viability to approximately 75%, and this effect plateaued at higher concentrations. Therefore, 10 μM of PD was selected for all subsequent in vitro experiments (Figure 4C). Then, Western blot analysis demonstrated that LPS stimulation markedly upregulated the protein levels of CitH3 and MPO compared to the control group. Co-treatment with PD significantly suppressed LPS-induced CitH3 and MPO expression (Figure 4D). Extracellular DNA release, a hallmark of NET formation, was assessed by SYTOX Green staining. LPS stimulation induced abundant extracellular DNA release, as evidenced by increased SYTOX Green fluorescence. PD treatment significantly attenuated this effect, reducing extracellular DNA release to near-control levels (Figure 4E). Furthermore, immunofluorescence co-staining of CitH3 and MPO was performed to visualize NET formation. In the control group, CitH3 and MPO co-localization was rarely observed (Figure 4F). LPS stimulation markedly increased the number of CitH3 and MPO double-positive cells, indicating robust NET formation. Consistent with the above findings, PD treatment significantly reduced the abundance of CitH3 and MPO double-positive cells compared to the LPS group (Figure 4F). Taken together, these in vitro results demonstrate that PD directly inhibits LPS-induced NET formation in neutrophils.

#### 3.1.5. PD Preserves Endothelial Barrier Integrity in a Neutrophil-Endothelial Co-Culture System

**Figure 5:**
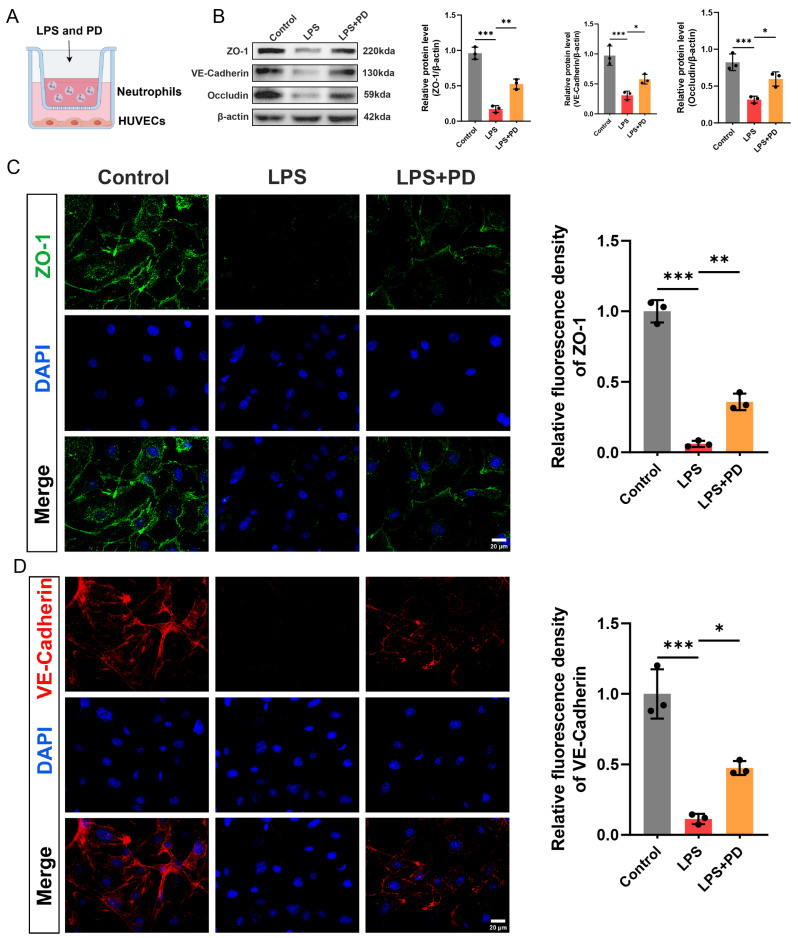
PD preserves endothelial barrier integrity in a neutrophil–endothelial co-culture system. (**A**) In vitro experimental protocol for this part. (**B**) Representative Western blots and quantitative analysis of ZO-1, VE–cadherin, and occludin protein levels in HUVECs (*n* = 3). (**C**) Representative immunofluorescence staining of ZO-1 (green) in HUVECs. Nuclei were counterstained with DAPI (blue), and quantitative analysis of ZO-1 fluorescence intensity (*n* = 3) was performed, scale bar = 20 μm. (**D**) Representative immunofluorescence staining of VE–cadherin (red) in HUVECs. Nuclei were counterstained with DAPI (blue), and quantitative analysis of VE–cadherin fluorescence intensity (*n* = 3) was performed, scale bar = 20 µm. * *p* < 0.05, ** *p* < 0.01, and *** *p* < 0.001.

To investigate whether PD protects against neutrophil-induced endothelial barrier disruption, we established a transwell co-culture system in which neutrophils were seeded in the upper chamber, and HUVECs were cultured in the lower chamber (Figure 5A). After 4 h of co-culture, HUVECs in the lower chamber were collected for analysis of tight junction and adherens junction protein expression. Western blot analysis revealed that LPS stimulation significantly downregulated the protein levels of ZO-1, VE–cadherin, and occludin in HUVECs compared to the control group. Co-treatment with PD significantly preserved the expression of all three junctional proteins (Figure 5B). To further visualize the integrity of endothelial junctions, immunofluorescence staining of ZO-1 and VE–cadherin was performed on HUVECs. ZO-1 immunostaining in the control group exhibited a continuous and intact pattern localized at cell–cell contacts. LPS stimulation disrupted this integrity, resulting in fragmented and diminished ZO-1 fluorescence intensity. PD treatment significantly restored ZO-1 fluorescence intensity and preserved its continuous distribution along the cell borders compared to the LPS group (Figure 5C). Similarly, VE–cadherin staining displayed a linear and continuous pattern along the endothelial cell borders in the control group, which was markedly disrupted by LPS stimulation, as evidenced by reduced and discontinuous staining. Consistent with the ZO-1 results, PD co-treatment significantly preserved VE–cadherin fluorescence intensity and maintained its junctional localization (Figure 5D). These results demonstrate that PD protects against neutrophil-mediated endothelial barrier disruption by preserving tight junction and adherens junction integrity in a co-culture system.

#### 3.1.6. PD Activates the Nrf2/HO-1 Pathway and Reduces ROS Production in Neutrophils

**Figure 6:**
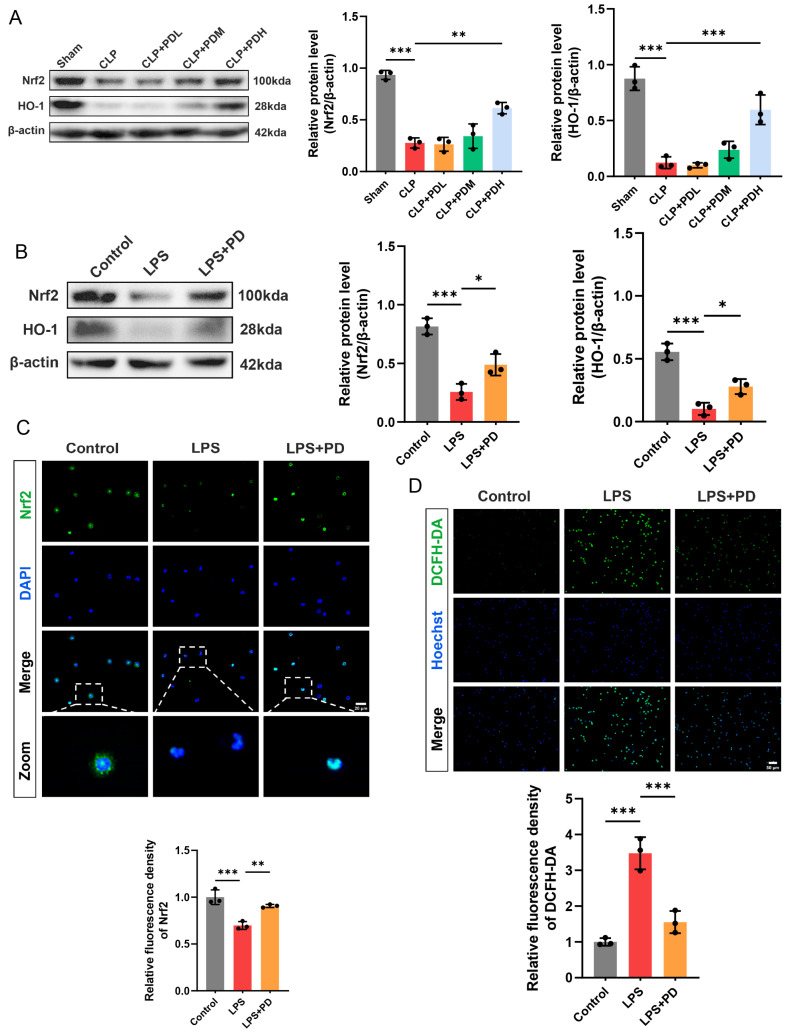
PD activates the Nrf2/HO-1 pathway and reduces ROS production in neutrophils. (**A**) Representative Western blots and quantitative analysis of Nrf2 and HO-1 protein levels in lung tissues (*n* = 3). (**B**) Representative Western blots and quantitative analysis of Nrf2 and HO-1 protein levels in neutrophils (*n* = 3). (**C**) Representative immunofluorescence staining of Nrf2 (green) in neutrophils. The nuclei were counterstained with DAPI (blue), and quantitative analysis of Nrf2 fluorescence intensity (*n* = 3) was performed, scale bar = 20 µm. (**D**) Representative immunofluorescence staining of intracellular ROS using DCFH-DA probe (green) in neutrophils. The nuclei were counterstained with DAPI (blue), and quantitative analysis of DCFH-DA fluorescence intensity (*n* = 3) was performed, scale bar = 50 µm. * *p* < 0.05, ** *p* < 0.01, and *** *p* < 0.001.

Given that NET formation is dependent on ROS production, we investigated whether PD suppresses NETosis by activating the Nrf2/HO-1 antioxidant pathway to attenuate oxidative stress in both in vivo and in vitro settings. Western blot analysis revealed that the CLP challenge significantly downregulated the protein levels of Nrf2 and HO-1 compared to the Sham group. PD treatment reversed this suppression in a dose-dependent manner. Notably, only the high dose of PD significantly increased Nrf2 and HO-1 expression compared to the CLP group (Figure 6A). To determine whether PD directly activates the Nrf2/HO-1 pathway in neutrophils, Western blot analysis demonstrated that LPS stimulation markedly reduced Nrf2 and HO-1 protein levels compared to the control group. PD co-treatment significantly upregulated both Nrf2 and HO-1 expression, restoring their levels toward those observed in the control group (Figure 6B). Immunofluorescence staining was performed to visualize Nrf2 subcellular localization. In control neutrophils, Nrf2 was predominantly localized in the cytoplasm, with moderate fluorescence intensity. LPS stimulation markedly diminished Nrf2 fluorescence intensity, with a weak signal observed in both cytoplasm and nucleus. PD treatment not only restored Nrf2 fluorescence intensity but also promoted its nuclear translocation, as evidenced by increased Nrf2 accumulation in the nucleus. Quantitative analysis confirmed that PD significantly increased total Nrf2 fluorescence intensity compared to the LPS group (Figure 6C). Consistent with the activation of the Nrf2/HO-1 antioxidant pathway, we next examined intracellular ROS levels using the DCFH-DA probe. LPS stimulation dramatically increased ROS production in neutrophils, as evidenced by enhanced green fluorescence. PD treatment significantly attenuated LPS-induced ROS generation, reducing fluorescence intensity to near-control levels (Figure 6D). These results demonstrate that PD activates the Nrf2/HO-1 antioxidant pathway and promotes Nrf2 nuclear translocation, thereby reducing LPS-induced ROS production in neutrophils.

#### 3.1.7. Nrf2 Inhibition Abrogates PD-Mediated Suppression of NET Formation in Neutrophils

**Figure 7:**
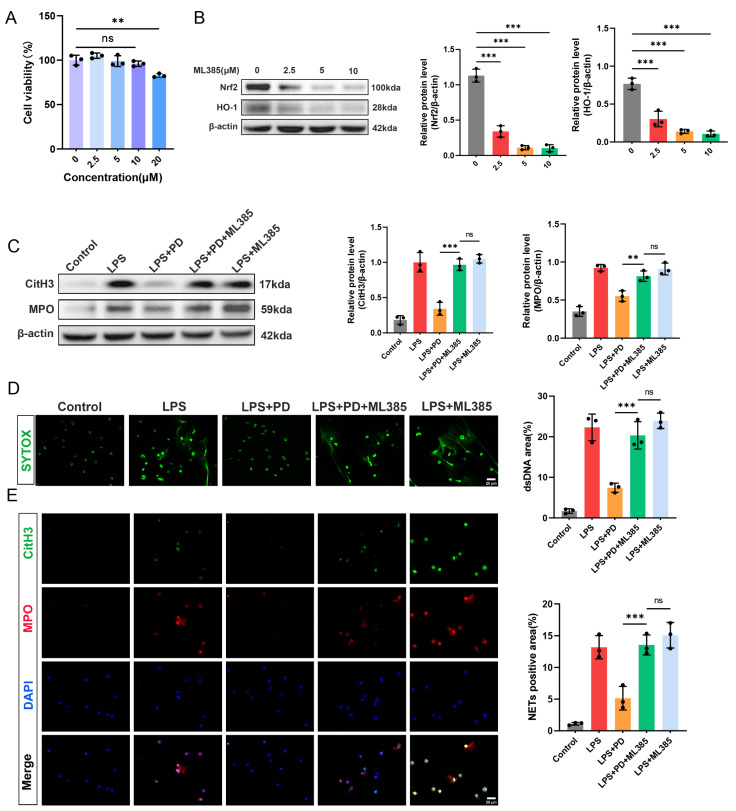
Nrf2 inhibition abrogates PD-mediated suppression of NET formation in neutrophils. (**A**) Neutrophils were treated with increasing concentrations of ML385 (0–20 μM) for 4 h, and cell viability was measured by CCK-8 assay (*n* = 3). (**B**) Representative Western blots and quantitative analysis of Nrf2 and HO-1 protein levels in neutrophils (*n* = 3). (**C**) Representative Western blots and quantitative analysis of CitH3 and MPO protein levels in neutrophils (*n* = 3). (**D**) Representative fluorescence images and quantitative analysis of SYTOX Green fluorescence intensity (*n* = 3), scale bar = 20 µm. (**E**) Representative immunofluorescence co-staining of CitH3 (green) and MPO (red) in neutrophils. Nuclei were counterstained with DAPI (blue), and quantitative analysis of CitH3 and MPO double-positive areas (*n* = 3) was performed, scale bar = 20 μm. ** *p* < 0.01, *** *p* < 0.001, and ns = not statistically significant.

To further confirm that PD inhibits NET formation through activation of the Nrf2/HO-1 pathway, we employed ML385, a specific Nrf2 inhibitor, in a series of in vitro experiments. First, to determine the optimal concentration of ML385 for subsequent experiments, neutrophils were treated with increasing concentrations of ML385 (0, 2.5, 5, 10, and 20 μM) for 4 h, and cell viability was assessed by CCK-8 assay. ML385 at concentrations ranging from 2.5 to 10 μM did not significantly affect neutrophil viability compared to the control. However, treatment with 20 μM ML385 significantly reduced cell viability to approximately 80% of the control level. Therefore, 10 μM ML385 was selected for all subsequent experiments to ensure effective Nrf2 inhibition without cytotoxicity (Figure 7A). Next, to validate the inhibitory effect of ML385 on the Nrf2/HO-1 pathway, neutrophils were treated with increasing concentrations of ML385 (0, 2.5, 5, and 10 μM), and the protein levels of Nrf2 and HO-1 were examined by Western blot. ML385 treatment dose-dependently suppressed the expression of both Nrf2 and HO-1. All three concentrations of ML385 significantly downregulated Nrf2 and HO-1 protein levels compared to the control group, with the most pronounced inhibition observed at 10 μM (Figure 7B). To determine whether Nrf2 activation is required for PD-mediated suppression of NET formation, we employed ML385 in LPS-stimulated neutrophils. The protective effect of PD against LPS-induced CitH3 and MPO upregulation was largely abrogated by the addition of ML385, as evidenced by significantly increased protein levels in the LPS + PD + ML385 group compared to the LPS + PD group. Notably, no significant difference was observed between the LPS + PD + ML385 group and the LPS + ML385 group, indicating that ML385 effectively blocked the protective effect of PD. Consistent with these findings, SYTOX Green staining revealed that ML385 reversed the inhibitory effect of PD on extracellular DNA release, with comparable fluorescence intensities observed between the LPS + PD + ML385 group and the LPS + ML385 group (Figure 7D). Similarly, immunofluorescence co-staining of CitH3 and MPO demonstrated that the reduction in NET-positive cells by PD was abolished by ML385 co-treatment, and no significant difference was detected between the LPS + PD + ML385 group and the LPS + ML385 group (Figure 7E). Taken together, these results demonstrate that PD suppresses LPS-induced NET formation, specifically through activation of the Nrf2/HO-1 pathway, as Nrf2 inhibition completely abrogates its protective effects.

## 4. Discussion

In this study, we demonstrated that PD attenuates sepsis-induced ALI by inhibiting NET formation. Mechanistically, we found that PD activates the Nrf2/HO-1 antioxidant pathway, thereby reducing ROS production and suppressing NETosis in neutrophils. These findings identify a novel mechanism by which PD protects against septic lung injury and highlight its therapeutic potential.

Neutrophils are key effector cells in the pathogenesis of septic ALI, while NETs are essential for pathogen containment. Excessive NET formation has been implicated in tissue damage, endothelial barrier disruption, and amplification of inflammatory responses during sepsis [33,34,35]. In the present study, we observed significant NET formation in the lungs of CLP-induced septic mice, as evidenced by increased Ly6G-positive neutrophil infiltration, elevated CitH3 and MPO expression, and enhanced extracellular DNA release. PD treatment dose-dependently suppressed these NET-associated parameters, with medium and high doses showing significant effects. These results are consistent with recent reports demonstrating that PD inhibits NET formation in other pathological conditions, including lupus, rheumatoid arthritis, and traumatic brain injury-induced lung injury. However, our study differs from these reports in several aspects. While Gu et al. showed that PD inhibits S100B-mediated NETs in traumatic brain injury [27], and Liao et al. and Yang et al. reported that PD reduces ROS-dependent NETs without identifying the upstream mechanism [28,29], our study identifies the Nrf2/HO-1 pathway as the specific mechanism by which PD inhibits NETs in sepsis, supported by causal validation using the Nrf2 inhibitor ML385. Thus, our findings provide the first causal evidence linking PD, Nrf2/HO-1 activation, and NET inhibition.

Endothelial barrier disruption is a hallmark of septic ALI, and NETs have been shown to directly injure pulmonary endothelial cells [36,37,38]. In this study, we found that PD preserved the expression of tight junction proteins and adherens junction proteins in lung tissues of the CLP mice, with medium and high doses demonstrating significant protective effects. Immunofluorescence staining further confirmed that PD maintained the continuous and intact distribution of these junctional proteins along endothelial cell borders. Using a transwell co-culture system, we demonstrated that PD protected against neutrophil-mediated endothelial injury by preserving endothelial junction integrity. These findings suggest that the inhibition of NET formation by PD translates into functional preservation of the endothelial barrier, thereby reducing pulmonary edema and vascular permeability observed in septic mice. It is important to note, however, that our assessment of endothelial barrier integrity relied primarily on molecular and morphological analyses. While our findings are consistent and robust, direct functional assays would provide an additional layer of validation. Future studies could incorporate Evans blue dye extravasation or FITC-dextran leakage assays to directly quantify vascular permeability, trans-endothelial electrical resistance (TEER) assays to assess endothelial barrier function in real time, and arterial blood gas analysis to evaluate pulmonary gas exchange in septic mice. Such functional endpoints would complement our molecular data and provide a more complete picture of PD’s therapeutic benefits.

The formation of NETs is critically dependent on intracellular ROS production [39,40,41]. The Nrf2/HO-1 pathway is a master regulator of antioxidant responses, and its activation has been shown to inhibit NETosis by reducing ROS levels in various disease models [42,43,44]. In this study, we observed that the CLP challenge significantly downregulated Nrf2 and HO-1 expression in lung tissues, which was reversed by high-dose PD treatment. In vitro, PD promoted Nrf2 nuclear translocation, upregulated HO-1 expression, and attenuated LPS-induced ROS production in neutrophils. These results indicate that PD activates the Nrf2/HO-1 antioxidant pathway to counteract oxidative stress during NET formation.

To establish a causal relationship between Nrf2/HO-1 activation and PD-mediated NET inhibition, we employed the specific Nrf2 inhibitor ML385 [45]. Notably, ML385 abrogated the inhibitory effect of PD on NET formation, as evidenced by restored CitH3 and MPO expression, increased extracellular DNA release, and enhanced NET-positive cells. Importantly, no significant difference was observed between the LPS + PD + ML385 group and the LPS + ML385 group, indicating that ML385 effectively blocked the protective effect of PD, and that PD exerts its inhibitory effect on NETosis specifically through the Nrf2/HO-1 pathway. While our in vitro experiments with the Nrf2 inhibitor ML385 established a causal link between Nrf2/HO-1 activation and PD’s anti-NETosis effects in isolated neutrophils, the in vivo validation of this mechanism remains an important area for future investigation. The correlative evidence from our animal model, showing that PD treatment upregulated Nrf2 and HO-1 expression in lung tissues strongly, suggests that this pathway is engaged in vivo. However, definitive proof would require genetic approaches, such as Nrf2-deficient mice or systemic administration of Nrf2 inhibitors in the CLP model. Such experiments would clarify whether the protective effects of PD are fully dependent on Nrf2/HO-1 activation in the whole animal or whether additional, parallel mechanisms contribute to its efficacy.

Several considerations should be considered when interpreting the findings of this study. First, while our study focused on neutrophils as the primary source of NETs, other cell types undoubtedly contribute to the pathogenesis of septic ALI. Alveolar epithelial cells are both targets of injury and active participants in the inflammatory response; they can release damage-associated molecular patterns that amplify inflammation and may also undergo NET-induced injury. Macrophages play a critical role in orchestrating the cytokine storm and can be polarized toward pro-inflammatory M1 or anti-inflammatory M2 phenotypes. Whether PD directly modulates macrophage polarization or protects alveolar epithelial cells from NET-induced injury remains to be determined. Future studies could address these questions using in vitro co-culture systems, such as alveolar epithelial cells or macrophages co-cultured with neutrophils, to assess cell-type-specific responses to PD. Additionally, in vivo cell depletion experiments, such as using clodronate liposomes to deplete macrophages, could help dissect the contribution of specific cell populations to PD’s protective effects. Second, the timing of PD administration in our study (1 h prior to CLP surgery) represents a preventive design that was appropriate for our mechanistic proof-of-concept investigation, as it allowed us to establish the causal relationship between PD, Nrf2/HO-1 activation, and NET inhibition with maximal drug exposure at the onset of sepsis. However, we fully acknowledge that this design does not recapitulate the clinical scenario, where treatment would typically be initiated after the onset of sepsis. To evaluate the true therapeutic potential of PD, future studies should employ delayed administration protocols, in which PD is administered at various time points after CLP surgery, such as 1 h, 3 h, or 6 h post-CLP, to mimic the clinical setting. Such studies would help determine the therapeutic window during which PD remains effective. Additionally, while our single-dose regimen was effective in this acute model, multiple-dose regimens may be required for sustained protection in a more clinically relevant context. Future studies should also investigate whether repeated dosing of PD, administered at intervals after CLP surgery, could enhance or prolong its protective effects. Systematic dose-response studies with delayed administration would help establish the optimal dosing strategy for potential clinical translation. Third, we did not evaluate systemic sepsis parameters or direct antimicrobial effects of PD; given that PD inhibited NETs, a key host defense mechanism, it is more plausible that PD acts by attenuating excessive inflammation rather than directly killing pathogens. Finally, the current study did not include a direct comparison with existing therapies, such as dexamethasone or sivelestat, which should be addressed in future translational studies.

This study demonstrates that polydatin alleviates sepsis-induced acute lung injury by inhibiting NET formation. In vitro mechanistic studies further suggest that this effect is mediated through activation of the Nrf2/HO-1 antioxidant pathway. These findings are significant for several reasons. First, we identify a novel mechanistic link between a natural compound and a key pathogenic process in sepsis. Second, we validate Nrf2/HO-1 as a critical regulatory node and therapeutic target for conditions characterized by excessive NETosis. Third, given its natural origin and favorable safety profile, PD represents a promising candidate for drug development. These findings provide new insights into the pathogenesis of septic ALI and lay the groundwork for future translational studies.

## 5. Conclusions

**Figure 8:**
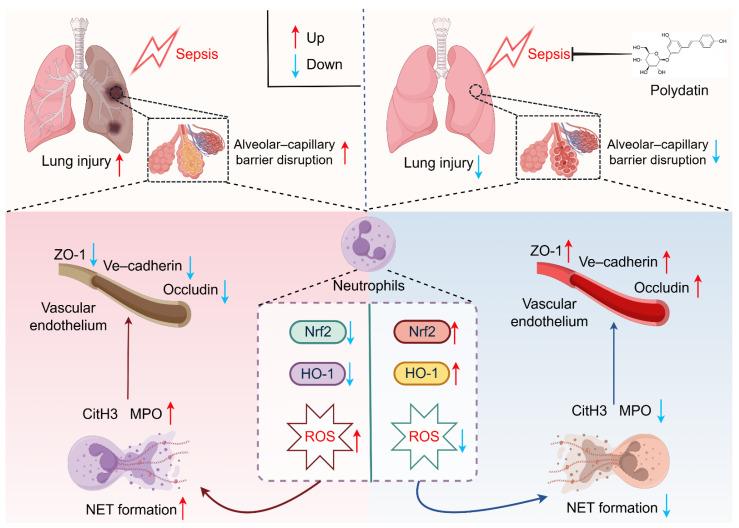
A schematic diagram of the proposed mechanism.

In summary, our study demonstrates that polydatin attenuates sepsis-induced acute lung injury by inhibiting NET formation through activation of the Nrf2/HO-1 antioxidant pathway, highlighting its potential as a therapeutic candidate for sepsis-associated lung injury (Figure 8).

## Figures and Tables

**Table 1:** Antibodies used in this study.

Antibody	Manufacturer	Catalog	Dilution
Anti-Histone H3 (citrulline R2 + R8 + R17) antibody	Abcam, Cambridge, UK	ab5103	1 µg/mL for WB 1:200 dilution for IF
Anti-MPO antibody	Abcam, Cambridge, UK	ab208670	1:1000 for WB 1:200 for IF
Anti-Ly6G antibody	Abcam, Cambridge, UK	ab238132	1:100 for IHC
Anti-ZO-1 antibody	Cell Signaling Technology, Danvers, MA, USA	13663	1:1000 for WB 1:200 for IF
Anti-VE cadherin antibody	Cell Signaling Technology, Danvers, MA, USA	60787	1:1000 for WB 1:200 for IF
Anti-occludin antibody	Proteintech, Rosemont, IL, USA	27260-1-AP	1:1000 for WB
Anti-Nrf2 antibody	Cell Signaling Technology, Danvers, MA, USA	12721	1:1000 for WB
Anti-HO-1 antibody	Cell Signaling Technology, Danvers, MA, USA	43966	1:1000 for WB
Anti-β-actin antibody	Proteintech, Rosemont, IL, USA	66009-1-lg	1:5000 for WB

**Table 2:** Reagents used in this study.

Reagents	Manufacturer	Catalog
Polydatin	Selleck, Houston, TX, USA	E7055
Lipopolysaccharide (LPS)	Sigma, St. Louis, MO, USA	L2630
ML385	MedChemExpress, Monmouth, NJ, USA	HY-100523
Mouse IL-6 ELISA kit	MultiSciences (Lianke) Biotech, Hangzhou, China	70-EK206
Mouse TNF-α ELISA kit	MultiSciences (Lianke) Biotech, Hangzhou, China	70-EK282
Mouse IL-1β ELISA kit	MultiSciences (Lianke) Biotech, Hangzhou, China	70-EK201B
Endothelial cell medium (ECM)	ScienCell, Carlsbad, CA, USA	1001
RPMI-1640	Gibco, Waltham, MA, USA	11875093
Fetal bovine serum (FBS)	Gibco, Waltham, MA, USA	10099141
Dimethyl sulfoxide (DMSO)	Sigma, St. Louis, MO, USA	D2650
Percoll	Solarbio, Beijing, China	P8370
SYTOX™ Green nucleic acid stain	Thermo Fisher Scientific, Waltham, MA, USA	60787
2′,7′-dichlorodihydrofluorescein diacetate (DCFH-DA)	Solarbio, Beijing, China	CA1410

## Data Availability

All data supporting the findings of this study are available within the article or from the corresponding author upon request.

## Supplementary Materials

The following supporting information can be downloaded at: https://www.mdpi.com/article/10.3390/biomedicines14040827/s1, Figure S1.PD improves 24-h survival and reduces clinical severity in septic mice.

## Abbreviations

The following abbreviations are used in this manuscript:

ALI	Acute lung injury
BALF	Bronchoalveolar lavage fluid
CitH3	Citrullinated histone H3
CLP	Cecal ligation and puncture
DCFH-DA	2′,7′-dichlorodihydrofluorescein diacetate
ELISA	Enzyme-linked immunosorbent assay
HO-1	Heme oxygenase-1
HUVECs	Human umbilical vein endothelial cells
LPS	Lipopolysaccharide
Ly6G	Lymphocyte antigen 6 complex locus G6D
MPO	Myeloperoxidase
NETs	Neutrophil extracellular traps
Nrf2	Nuclear factor erythroid 2-related factor 2
ROS	Reactive oxygen species
VE–cadherin	Vascular endothelial cadherin
ZO-1	Zonula occludens-1
