# Supplementary material for: Polydatin Attenuates Sepsis-Induced Lung Injury by Inhibiting Neutrophil Extracellular Traps Formation via Nrf2/HO-1 Pathway
Source: Biomedicines. 2026 Apr 5;14(4):827. doi: 10.3390/biomedicines14040827 (PMC13113620; doi:10.3390/biomedicines14040827)
Supplement: Supplementary file 1 [file biomedicines-4207267-supplementary.pdf › biomedicines-4207267-supplementary.pdf]

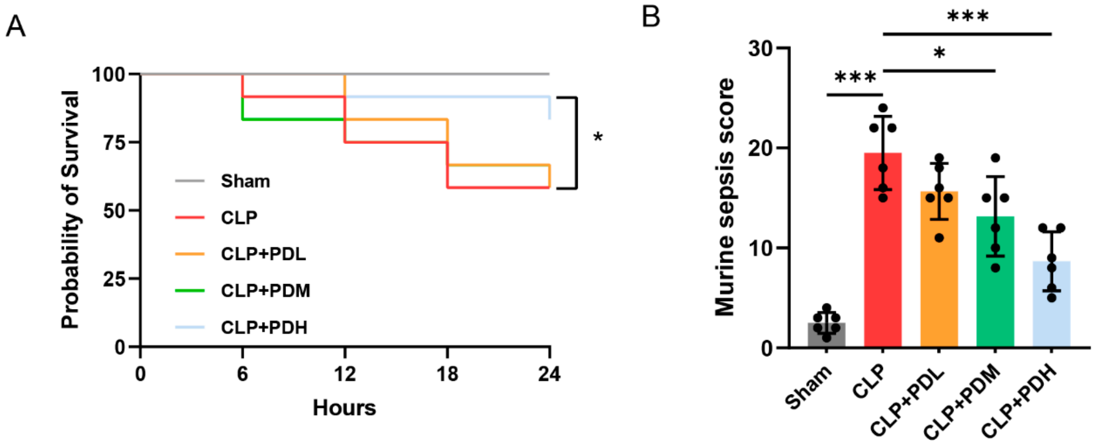

Figure S1. PD improves 24-hour survival and reduces clinical severity in septic mice. (**A**) the 24-hour survival rate of mice in each group was observed every 6 h (Sham group, n = 6; other groups, n = 12). (**B**) MSS at 24 hours post-surgery (n = 6). * $p < 0.05$, ** $p < 0.01$, *** $p < 0.001$.
